# Antiproliferation of *Cryptocarya concinna*-derived cryptocaryone against oral cancer cells involving apoptosis, oxidative stress, and DNA damage

**DOI:** 10.1186/s12906-016-1073-5

**Published:** 2016-03-08

**Authors:** Hsun-Shuo Chang, Jen-Yang Tang, Ching-Yu Yen, Hurng-Wern Huang, Chang-Yi Wu, Yi-An Chung, Hui-Ru Wang, Ih-Sheng Chen, Ming-Yii Huang, Hsueh-Wei Chang

**Affiliations:** Graduate Institute of Natural Products, College of Pharmacy, Kaohsiung Medical University, Kaohsiung, Taiwan; School of Pharmacy, College of Pharmacy, Kaohsiung Medical University, Kaohsiung, Taiwan; Department of Radiation Oncology, Faculty of Medicine, College of Medicine, Kaohsiung Medical University, Kaohsiung, Taiwan; Department of Radiation Oncology, Kaohsiung Medical University Hospital, Kaohsiung, Taiwan; Department of Radiation Oncology, Kaohsiung Municipal Ta-Tung Hospital, Kaohsiung, Taiwan; Department of Oral and Maxillofacial Surgery Chi-Mei Medical Center, Tainan, Taiwan; School of Dentistry, Taipei Medical University, Taipei, Taiwan; Institute of Biomedical Science, National Sun Yat-Sen University, Kaohsiung, Taiwan; Department of Biological Sciences, National Sun Yat-Sen University, Kaohsiung, Taiwan; Department of Biomedical Science and Environmental Biology, Kaohsiung Medical University, Kaohsiung, Taiwan; Institute of Medical Science and Technology, National Sun Yat-Sen University, Kaohsiung, Taiwan; Cancer Center, Kaohsiung Medical University Hospital, Kaohsiung Medical University, Kaohsiung, Taiwan; Center for Reseach Resources and Development of Kaohsiung Medical University, Kaohsiung, Taiwan

**Keywords:** *Cryptocarya concinna*, Cryptocaryone, Oral cancer, Apoptosis, Oxidative stress, γH2AX

## Abstract

**Background:**

*Cryptocarya*-derived crude extracts and their compounds have been reported to have an antiproliferation effect on several types of cancers but their impact on oral cancer is less well understood.

**Methods:**

We examined the cell proliferation effect and mechanism of *C. concinna*-derived cryptocaryone (CPC) on oral cancer cells in terms of cell viability, apoptosis, reactive oxygen species (ROS), mitochondrial depolarization, and DNA damage.

**Results:**

We found that CPC dose-responsively reduced cell viability of two types of oral cancer cells (Ca9-22 and CAL 27) in MTS assay. The CPC-induced dose-responsive apoptosis effects on Ca9-22 cells were confirmed by flow cytometry-based sub-G1 accumulation, annexin V staining, and pancaspase analyses. For oral cancer Ca9-22 cells, CPC also induced oxidative stress responses in terms of ROS generation and mitochondrial depolarization. Moreover, γH2AX flow cytometry showed DNA damage in CPC-treated Ca9-22 cells. CPC-induced cell responses in terms of cell viability, apoptosis, oxidative stress, and DNA damage were rescued by N-acetylcysteine pretreatment, suggesting that oxidative stress plays an important role in CPC-induced death of oral cancer cells.

**Conclusions:**

CPC is a potential ROS-mediated natural product for anti-oral cancer therapy.

**Electronic supplementary material:**

The online version of this article (doi:10.1186/s12906-016-1073-5) contains supplementary material, which is available to authorized users.

## Background

Oral cancer is the sixth most common cancer in the world [1, 2]. Oral cancer is easy to detect clinically but is frequently ignored by patients resulting in high mortality rates [3]. Although several oral tumor markers have been reported [4, 5], these efforts focused on detection rather than therapy and effective anti-oral cancer therapies are still needed.

Cryptocarya (family Lauraceae) is widely found in the tropics and subtropics [6]. Its crude extracts have been demonstrated to have an antiproliferative ability against cancer. For example, methanolic extracts of the leaves of *C. griffithiana* and the roots of *C. concinna* can inhibit cell proliferation of human HL60 promyelocytic leukemia cells [7] and oral cancer cells [8], respectively. The ethanolic extracts of the fruit and trunk bark of *C. obovata* have been reported to have an antiproliferative effect against human KB cells [9].

Several *Cryptocarya*-derived compounds have been found to have diverse biological functions, i.e., anti-dengue virus by alkylated flavanones [10], anti-HIV by phenanthroindolizidine alkaloids [11], anti-tuberculosis by pinocembrin [12], anti-plasmodial by (+)-N-methylisococlaurine, atherosperminine, and 2-hydroxy-atherosperminine [13], anti-trypanosomal by 7′,8′-dihydroobolactone [14], and anti-inflammatory by (2S)-5,7-dihydroxyflavanone and cryptocaryanone B [15].

Recently, *Cryptocarya*-derived compounds have been reported to have an antiproliferation effect on cancer. For example, the proliferation of leukemia cells was inhbitied by *C. costata*-derived 2′,4′-dihydroxy-5′,6′-dimethoxychalcone and isodidymocarpin [16] and *C. konishii*-derived by desmethylinfectocaryone, infectocaryone, and cryptocaryone (CPC) [17]. Among these *Cryptocarya*-derived compounds, we are interested in the anticancer effect of CPC, which is one of the major constituents in the commonly distributed evergreen plant *C. concinna* in Taiwan [18].

Although the anticancer effect of CPC, a kind of dihydrochalcone, had been reported in some cancer types such as murine leukemia [17] and prostate cancer [19], few studies have addressed its antiproliferative effect on oral cancer. Moreover, the cell killing mechanism of cryptocaryone in cancer remains unclear. Recently, reactive oxygen species (ROS) generation was reported to be involved in *Corema album*-derived dihydrochalcone induced cytotoxicity for colon cancer cells [20]. Accordingly, the relationship between ROS generation and CPC effect for oral cancer cells is worth examining.

This study evaluates possible anticancer functions of CPC and explores its drug mechanisms in terms of cell viability, cell cycle analysis, apoptosis, ROS generation, mitochondrial depolarization, and DNA damage detection. The role of oxidative stress in CPC’s effect on oral cancer cells is also addressed.

## Methods

### Plant material and isolation

*C. concinna* was identified by one of the authors (Ih-Sheng Chen) and its roots were collected at Mudan, Pingtung County, Taiwan, in May 2004. A voucher specimen (Chen6153) has been deposited in the Herbarium of the School of Pharmacy, College of Pharmacy, Kaohsiung Medical University. The dried roots (7.7 Kg) of *C. concinna* were processed by slicing and cold methanol-extraction three times at room temperature. Finally, the solution was evaporated under reduced pressure to yield the methanolic extract (800 g; yield: methanolic extract/dried roots = 10.4 %) [8]. CPC (5.7 g; yield: CPC/methanolic extract = 0.7 %) was isolated from the root of *C. concinna* as described previously [19]. In brief, the methanolic extract was partitioned between chloroform/water (1:1) to yield a chloroform fraction and a water fraction. The chloroform fraction was subjected to silica gel column chromatography and eluted with a gradient of chloroform–methanol to produce 13 fractions (A-1–A-13). CPC was then obtained from fraction A-3 (chloroform-methanol 100:1) and the structure of CPC was determined by spectral analyses (Additional file 1).

### Cell cultures and chemicals

Two human oral cancer cell lines (Ca9-22 [21] and CAL 27 [22]), purchased from the Cell Bank, RIKEN BioResource Center (Tsukuba, Japan) and the American Type Culture Collection (ATCC; Virginia, USA), respectively, were incubated in DMEM/F12 (3:2) medium (Gibco, Grand Island, NY, USA) supplemented with 10 % fetal bovine serum (Gibco), 100 U/ml penicillin, 100 μg/ml streptomycin, and 0.03 % glutamine. Normal gingival fibroblast (HGF-1) was purchased from ATCC and maintained in DMEM medium (Gibco, Grand Island, NY, USA) with a similar supplement with 1 mM pyruvate as described above. Cells were incubated in humidified air at 37 °C with 5 % CO_2_. N-acetylcystein (NAC) was purchased from Sigma (St. Louis, MO, USA) for pretreatment before CPC application. Passage numbers of the oral cancer (Ca9-22 and CAL 27) cells used in this study were 15–22 and 8–15, respectively.

### Cell viability

Cell viability was determined using the CellTiter 96® AQueous One Solution Cell Proliferation Assay (MTS) (Promega Corporation, Madison,WI, USA) as previously described [21]. Two oral cancer cell lines (Ca9-22 and CAL 27) were seeded at 1 × 10^5^ cells per well and HGF-1 cells were seeded at 4 × 10^4^ cells per well in a 6-well plate, respectively. After seeding for 24 h, cells were treated with CPC at indicated concentrations for 24 h and cell viability was determined by an ELISA reader at 490 nm.

### Determination of cell cycle distribution

Propidium iodide (PI) (Sigma, St Louis, MO, USA) was added to stain the cellular DNA content [23]. In brief, 3 × 10^5^ cells per well in 6 well plates were seeded overnight and then treated with the vehicle (0.05 % DMSO) or 3, 6, 9, 12 μM of CPC for 24 h. After cells were harvested and washed twice with PBS, they were fixed overnight with 70 % ethanol. Subsequently, the cell pellets were resuspended in 50 μg/ml PI for 30 min at 37 °C in darkness. The cell cycle distribution was evaluated by a flow cytometer (BD Accuri™ C6; Becton-Dickinson, Mansfield, MA, USA) and BD Accuri™ C6 software (version 1.0.264).

### Determination of apoptosis by annexin V/PI

Apoptosis was detected by annexin V (Strong Biotect Corporation, Taipei, Taiwan)/PI (Sigma, St Louis, MO, USA) as described in [24]. Briefly, 3 × 10^5^ cells per well in 6 well plates were seeded for 24 h and then treated with the vehicle or indicated concentrations of CPC for 24 h. Cells were then incubated with 100 μl binding buffer containing 2 μl of annexin-V-fluorescein isothiocyanate (FITC) stock (0.25 μg/μl) and 2 μl of PI stock (1 mg/ml) for 30 min. Finally, it was suspended with 400 μl PBS for flow cytometry analysis (BD Accuri™ C6; Becton-Dickinson).

### Determination of apoptosis by pancaspase activity

The apoptosis was also detected by the measurement of caspase activation [25]. In this study, the generic activation of pancaspases (Caspase-1, 3, 4, 5, 6, 7, 8, 9) was determined by the generic caspase activity assay kit (Abcam, Cambridge, UK) as described in [26]. Briefly, Ca9-22 cells were seeded as 3 × 10^5^ cells per well in 6 well plates with 2 ml medium. The next day, Ca9-22 cells were treated with CPC for 24 h, 2 μl of 500X TF2-VAD-FMK was then added, and the cells were incubated at 37 °C, 5 % CO_2_ for 2 h. Cells were washed with PBS twice and resuspended in 0.5 ml of assay buffer for immediate flow cytometry measurement (BD Accuri™ C6; Becton-Dickinson).

### Determination of intracellular ROS

The dye 2′,7′-dichlorodihydrofluorescein diacetate (DCFH-DA) was used to detect ROS by its fluorescence change [27]. Cells at the density of 3 × 10^5^ in 2 ml medium per well in 6 well plates were seeded for 24 h. Different concentrations of CPC were added to Ca9-22 cells for 3 h. After washing with PBS, 100 nM DCFH-DA in PBS were added to the cells in 6 well plates in a cell culture incubator for 30 min. After trypsinization, PBS washing, and centrifugation, cell pellets were resuspended in 1 ml PBS before flow cytometry analysis (BD Accuri™ C6; Becton-Dickinson) and its software.

### Determination of mitochondrial membrane potential (MitoMP)

MitoProbe™ DiOC_2_(3) assay kit (Invitrogen, Eugene, OR, USA) was applied to analyze MitoMP as described previously [28, 29]. Briefly, 3 × 10^5^ cells in 2 ml medium per well in 6 well plates were seeded for 24 h. After CPC treatment, 10 μl of 10 μM DiOC_2_(3) was added per well and incubated in a cell culture incubator for 30 min. After harvesting, cells were resuspended in 1 ml PBS for flow cytometry analysis (BD Accuri™ C6; Becton-Dickinson).

### Determination of DNA damage by γH2AX/PI

DNA double strand breaks were detected by flow cytometry as described previously [30]. In brief, CPC-treated cells were fixed in 70 % ethanol, washed with BSA-T-PBS solution (1 % bovine serum albumin and 0.2 % Triton X-100 in PBS; Sigma), and incubated at 4 °C for 1 h in 100 μl of BSA-T-PBS solution containing 0.2 μg p-Histone H2A.X (Ser 139) monoclonal antibody (Santa Cruz Biotechnology, Santa Cruz, CA, USA). Cells were washed and suspended for 1 h in a 1:50 dilution of Alexa Fluor 488-tagged secondary antibody (Jackson Laboratory, Bar Harbor, ME, USA) for staining for 30 min at room temperature. Finally, the cells were resuspended in 20 μg/ml of PI for flow cytometry analysis (BD Accuri™ C6; Becton-Dickinson).

### Statistical analysis

Group differences of the same drug treatment with different concentrations were analyzed by one-way ANOVA with Tukey HSD Post Hoc Test using JMP® 10 software [21]. No overlapping by the same lower-case letter indicated significant differences.

## Results

### Assessment of antiproliferation of CPC and the effect of NAC pretreatment

**Fig. 1:**
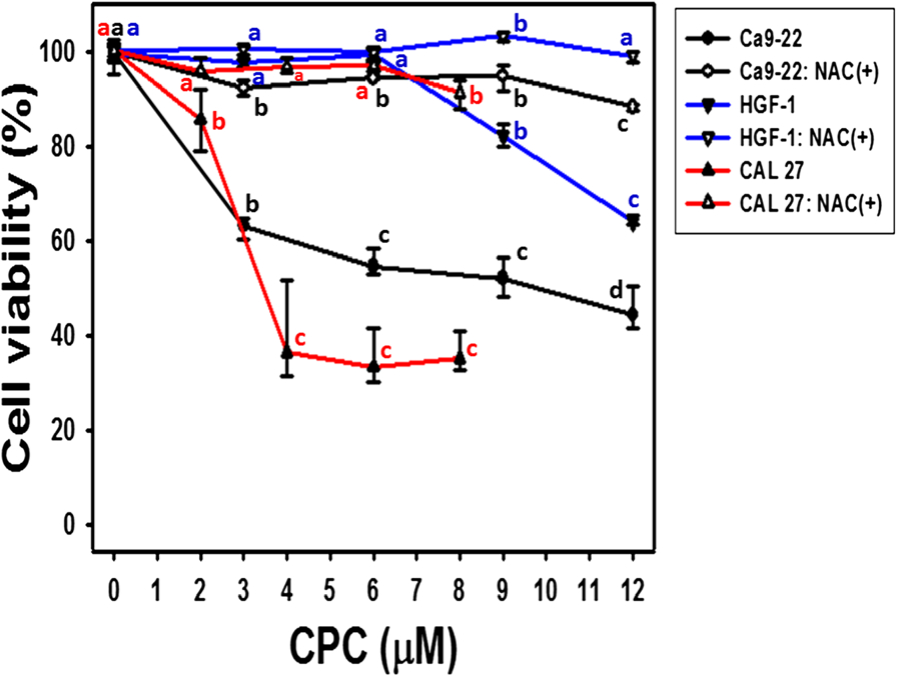
Cell viabilities of CPC-treated oral cancer cells and the effect of NAC pretreatment. Oral cancer (Ca9-22 and CAL 27) cells and oral normal (HGF-1) cells were chosen. With or without 2 mM NAC pretreatment for 1 h, Ca9-22 and HGF-1 cells were treated with 3, 6, 9, and 12 μM of CPC and CAL 27 cells were treated with 2, 4, 6, and 8 μM of CPC for 24 h incubation. The cell viability was measured by the MTS assay. Data, means ± SDs (*n* = 6). For the same drug treatment of different concentrations, data marks (a to d) without overlapping by the same lower-case letter significantly differed (one-way ANOVA with Tukey HSD Post Hoc Test)

In the MTS assay (Fig. 1), the cell viability (%) of two oral cancer cells (Ca9-22 and CAL 27) at indicated concentrations of CPC were dose-responsively decreased (*P* < 0.05–0.001 compared to the vehicle). In contrast, the cell viabilities of oral normal HGF-1 cells were similar to the control at 3 and 6 μM of CPC although cell viability decreased at 9 and 12 μM. The IC_50_ values of CPC for oral cancer Ca9-22 and CAL 27 cells were 9.87 and 3.45 μM, respectively, whereas IC_50_ was undetected in the CPC-treated oral normal HGF-1 cells under the same CPC treatment.

NAC, an ROS scavenger, is commonly used to validate the role of oxidative stress in drug-induced ROS-mediated effects [31–34]. NAC pretreatment was performed to examine the possible role of oxidative stress in CPC-induced cell death, showing that the CPC-induced antiproliferation in oral cancer cells and normal cells were almost completely rescued compared to controls (Fig. 1).

### Assessment of sub-G1 accumulation of CPC and the effect of NAC pretreatment

**Fig. 2:**
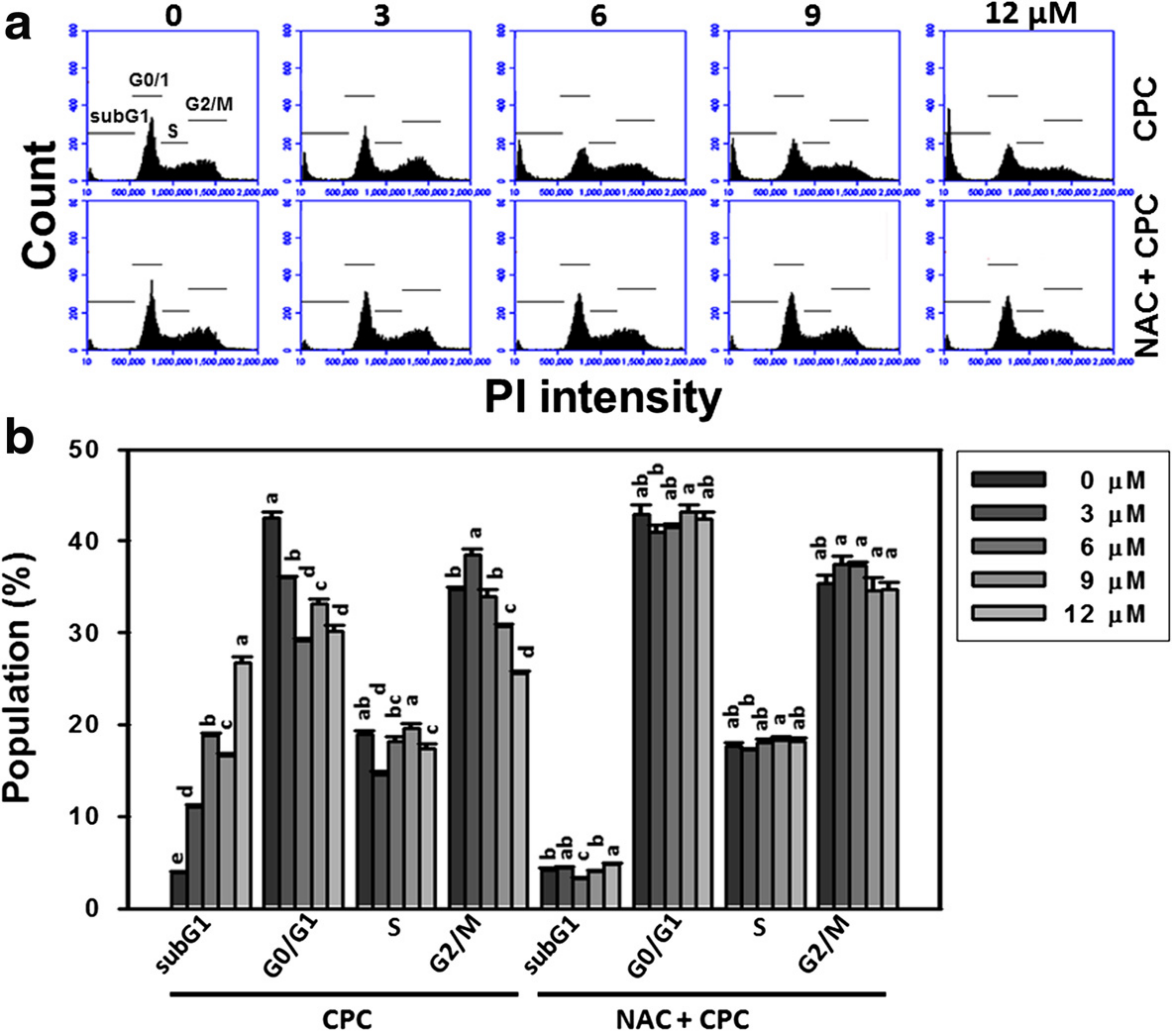
The sub-G1 accumulations of CPC-treated oral cancer Ca9-22 cells and the effect of NAC pretreatment. With or without 2 mM NAC pretreatment for 1 h, Ca9-22 cells were treated with 0, 3, 6, 9, and 12 μM of CPC for 24 h. **a** Representative flow cytometry-based cell cycle distribution profiles of CPC-treated Ca9-22 cells. The cell cycle phases are underlined in each panel. **b** Quantification analysis for the subG1 percentages in Fig. 2a. Data, mean ± SD (*n* = 3). For the same drug treatment of different concentrations, data marks (a to d) without overlapping by the same lower-case letter significantly differed (one-way ANOVA with Tukey HSD Post Hoc Test)

The distribution profiles of CPC-induced cell cycle changes of oral cancer Ca9-22 cells are shown in Fig. 2a (top). After CPC treatment (Fig. 2b), the sub-G1 populations of CPC (0–12 μM)-treated Ca9-22 cells were dose-responsively increased (*P* < 0.001). In contrast, the G0/G1 and G2/M populations of CPC-treated Ca9-22 cells were almost dose-responsively decreased (*P* < 0.001).

The distribution profiles of NAC pretreatment effect against CPC-induced cell cycle changes are shown in Fig. 2a (bottom). After NAC pretreatment, i.e., NAC + CPC (Fig. 2b, right), the CPC-induced cell cycle changes as mentioned above recovered to normal distributions compared to CPC only (Fig. 2b, left) and untreated controls.

### Assessment of annexin V/PI-based apoptosis of CPC and the effect of NAC pretreatment

**Fig. 3:**
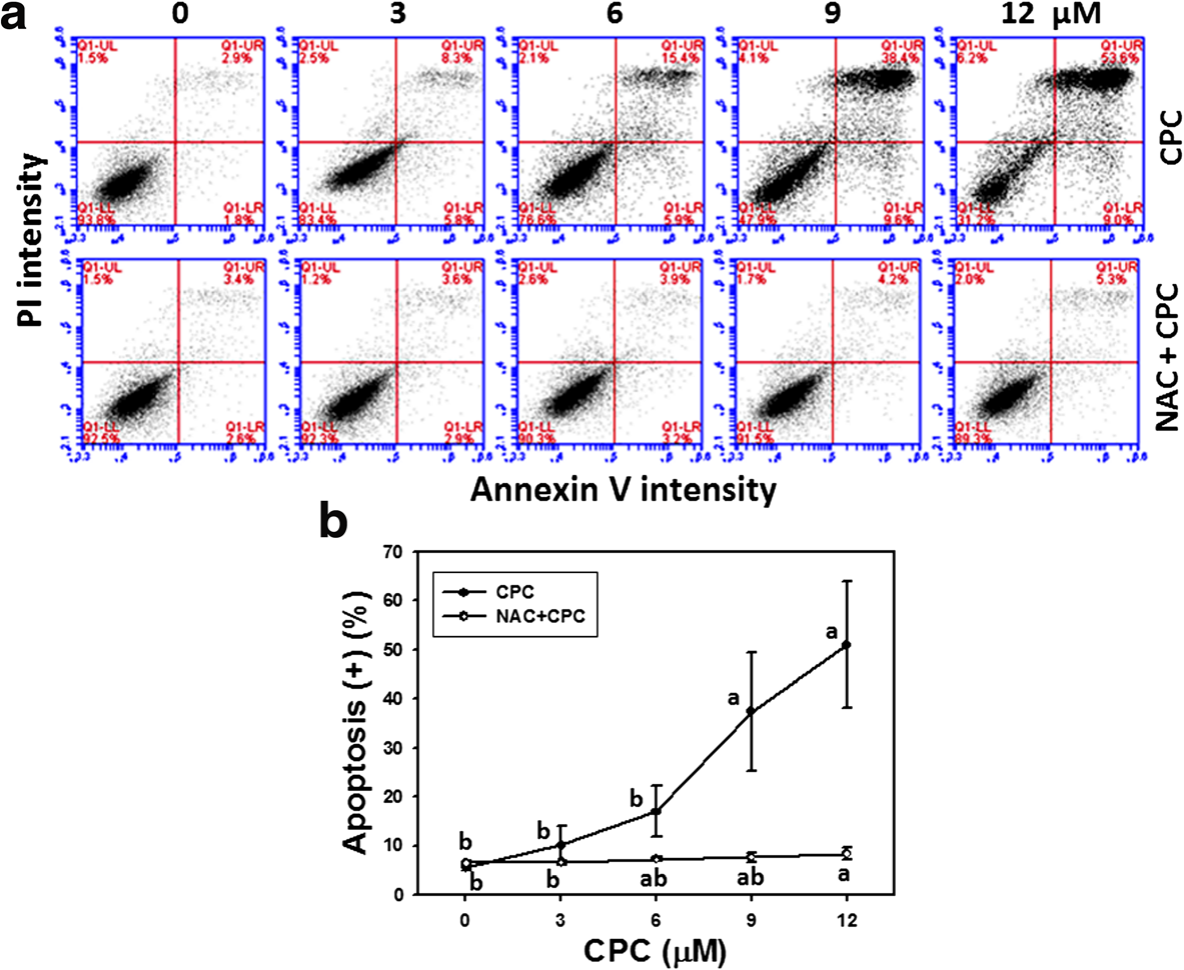
Annexin V/PI-based apoptosis of CPC-treated oral cancer Ca9-22 cells and the effect of NAC pretreatment. With or without 2 mM NAC pretreatment for 1 h, Ca9-22 cells were treated with 0, 3, 6, 9, and 12 μM of CPC for 24 h. **a** Representative results of flow cytometry-based annexin V/PI double staining of CPC-treated Ca9-22 cells. Annexin V (+)/PI (+) and Annexin V (+)/PI (-) were calculated as the apoptosis (+) in each panel. **b** Quantification analysis of apoptosis for CPC-treated Ca9-22 cells in Fig. 3a. Data, mean ± SD (*n* = 3). For the same drug treatment of different concentrations, data marks (a and b) without overlapping by the same lower-case letter significantly differed (one-way ANOVA with Tukey HSD Post Hoc Test)

To further validate the role of apoptosis, the annexin V/PI profiles of CPC-treated oral cancer Ca9-22 cells were demonstrated by flow cytometry (Fig. 3a, top). In Fig. 3b, the populations of annexin V-positive intensities for CPC (0–12 μM)-treated Ca9-22 cells were dose-responsively increased (*P* < 0.05–0.001).

The annexin V/PI profiles of NAC pretreatment effect against CPC-induced apoptosis were also demonstrated in Fig. 3a (bottom). After NAC pretreatment, i.e., NAC + CPC (Fig. 3b), the CPC-induced apoptosis changes as mentioned above recovered to normal levels compared to CPC only and untreated controls.

### Assessment of pancaspase-based apoptosis of CPC and the effect of NAC pretreatment

**Fig. 4:**
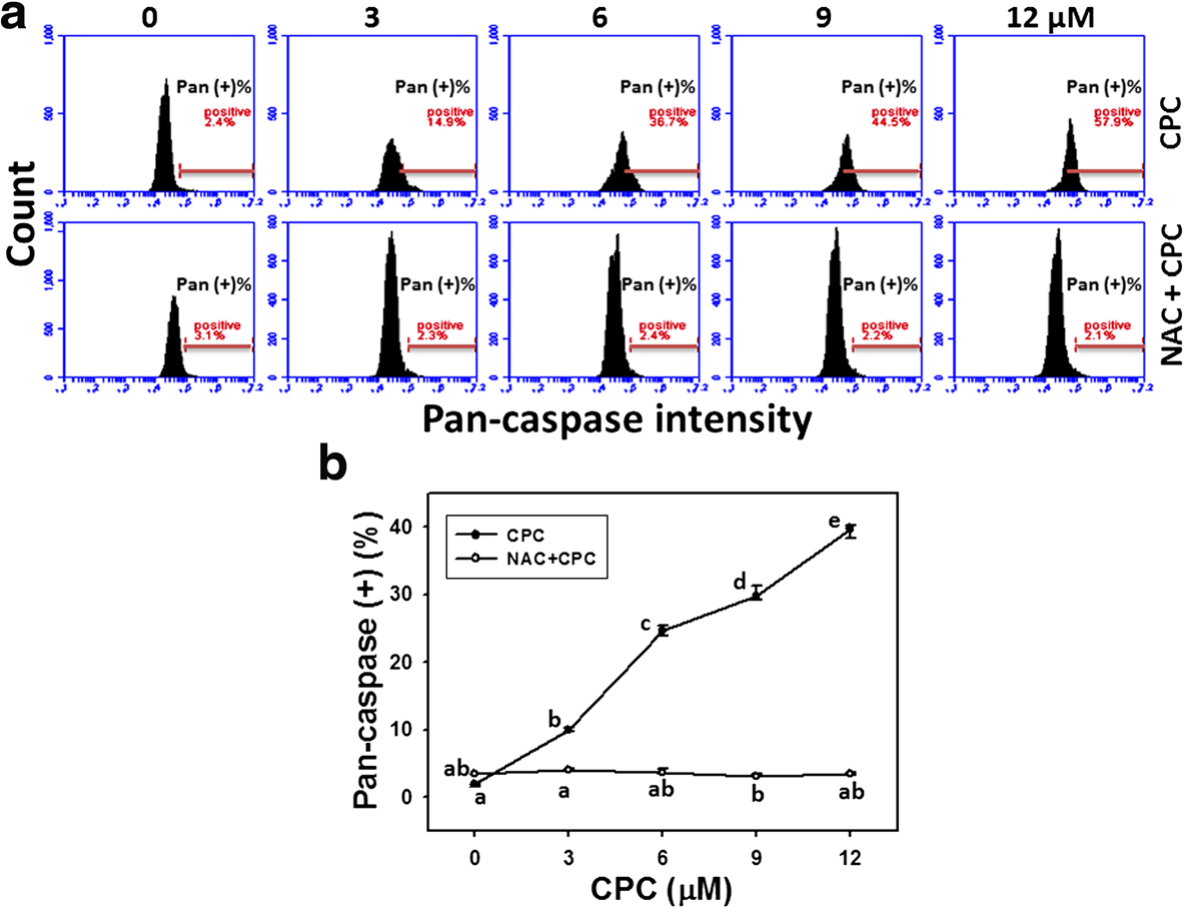
Generic caspase-based apoptosis of CPC-treated oral cancer Ca9-22 cells and the effect of NAC pretreatment. **a** With or without 2 mM NAC pretreatment for 1 h, Ca9-22 cells treated with 0, 3, 6, 9, and 12 μM of CPC for 24 h were stained with 1 μl 500X TF2-VAD-FMK. The right side indicates the pancaspase (Pan) positive region in each panel. **b** Quantificative analysis of pan-caspase fluorescent intensity. Data, mean ± SD (*n* = 3). For the same drug treatment of different concentrations, data marks (a to e) without overlapping by the same lower-case letter significantly differed (one-way ANOVA with Tukey HSD Post Hoc Test)

To further validate the role of apoptosis, the pancaspase profiles of CPC-treated oral cancer Ca9-22 cells were demonstrated by flow cytometry (Fig. 4a, top). In Fig. 4b, the populations of pancaspase-positive intensities for CPC (0–12 μM)-treated Ca9-22 cells were dose-responsively increased (*P* < 0.001).

The pancaspase profiles of NAC pretreatment effect against CPC-induced apoptosis were also demonstrated in Fig. 4a (bottom). After NAC pretreatment, i.e., NAC + CPC (Fig. 4b), the CPC-induced apoptosis changes as mentioned above recovered to normal levels compared to CPC only and untreated controls.

### Assessment of ROS generation of CPC and the effect of NAC pretreatment

**Fig. 5:**
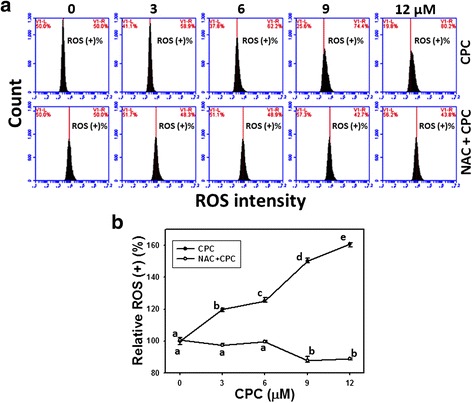
ROS generation of CPC-treated oral cancer Ca9-22 cells and the effect of NAC pretreatment. With or without 2 mM NAC pretreatment for 1 h, Ca9-22 cells were treated with 0, 3, 6, 9, and 12 μM of CPC for 6 and 12 h. **a** Representative ROS profiles of flow cytometry for CPC treated Ca9-22 cells. The right side indicates the ROS positive region in each panel. **b** Quantification analysis of relative ROS intensity in Fig. 5a. Data, mean ± SD (*n* = 3). For the same drug treatment of different concentrations, data marks (a to e) without overlapping by the same lower-case letter significantly differed (one-way ANOVA with Tukey HSD Post Hoc Test)

Accumulated evidence showed that ROS-generating drugs and natural products may lead to apoptosis [35–38]. To validate the role of ROS in the CPC induced cell death effect, an ROS staining dye (DCFH-DA) was applied to flow cytometry. Figure 5a shows the ROS staining profiles of CPC-treated Ca9-22 cells at 3 h incubation. After CPC treatment, the relative ROS-positive staining of CPC (0–12 μM)-treated Ca9-22 cells were dose-responsively induced (*P* < 0.001) (Fig. 5b).

The ROS staining profiles of NAC pretreatment effects against CPC-induced ROS generation were also demonstrated in Fig. 5a (bottom). After NAC pretreatment, i.e., NAC + CPC (Fig. 5b), the CPC-induced ROS changes as mentioned above recovered to normal levels compared to CPC only and untreated controls.

### Assessment of MitoMP of CPC and the effect of NAC pretreatment

**Fig. 6:**
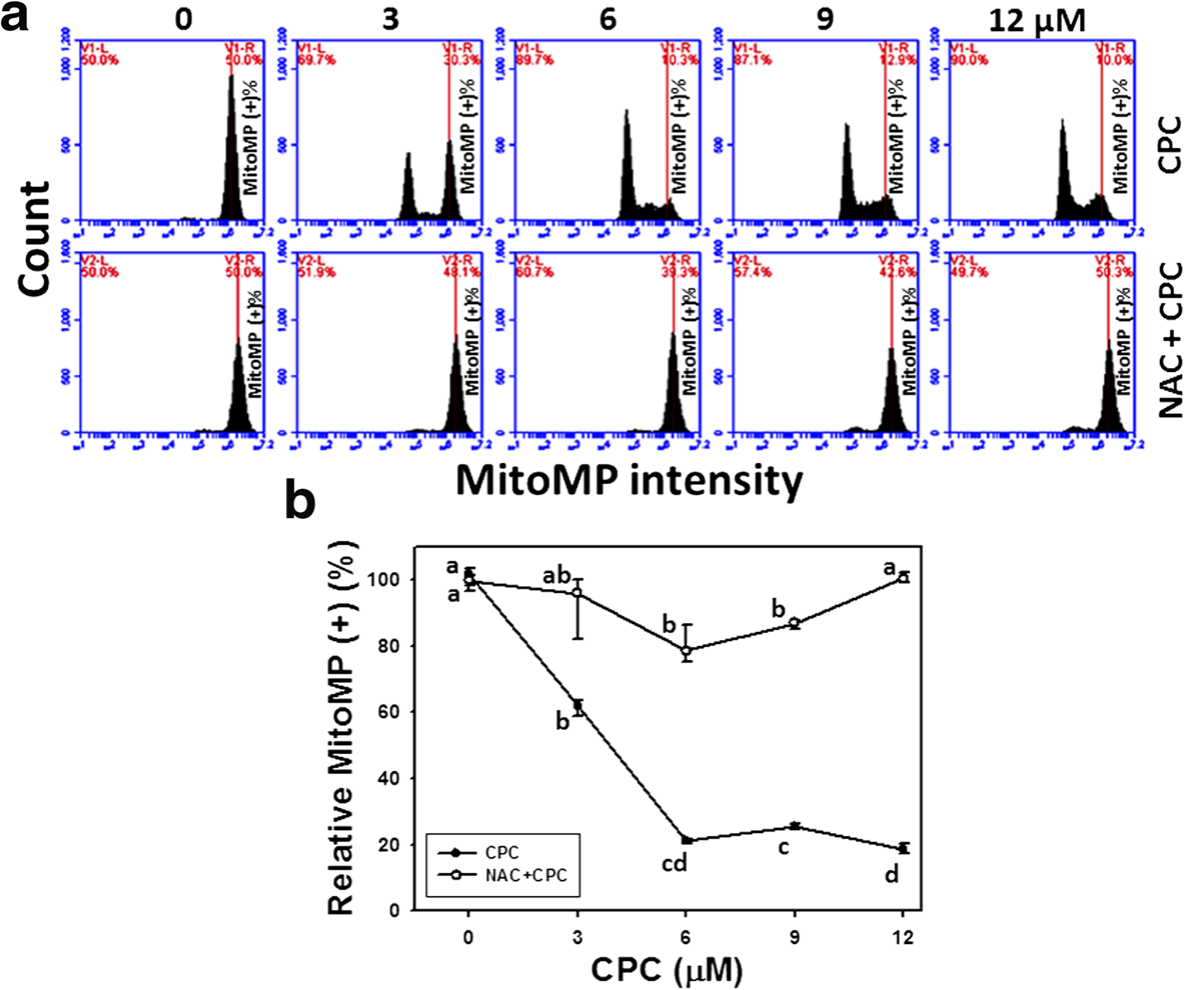
Depolarization of mitochondrial membrane potential (MitoMP) of CPC-treated oral cancer Ca9-22 cells and the effect of NAC pretreatment. With or without 2 mM NAC pretreatment for 1 h, Ca9-22 cells were treated with 0, 3, 6, 9, and 12 μM of CPC for 24 h. **a** Representative MitoMP profiles of flow cytometry for CPC treated Ca9-22 cells. The right side indicates the MitoMP positive region in each panel. **b** Quantification analysis of relative MitoMP intensity in Fig. 6a. Data, mean ± SD (*n* = 3). For the same drug treatment of different concentrations, data marks (a to d) without overlapping by the same lower-case letter significantly differed (one-way ANOVA with Tukey HSD Post Hoc Test)

To validate the role of MitoMP in the CPC-induced effects, a mitochondrial membrane potential-sensitive staining dye (DiOC_2_(3)) was applied to flow cytometry. Figure 6a shows the MitoMP staining profiles of CPC-treated Ca9-22 cells at 24 h incubation. After CPC treatment, the relative MitoMP-positive staining of CPC (0–12 μM)-treated Ca9-22 cells were dose-responsively decreased (*P* < 0.001) (Fig. 6b).

The MitoMP staining profiles of NAC pretreatment effects against CPC-induced ROS generation were also demonstrated in Fig. 6a (bottom). After NAC pretreatment, i.e., NAC + CPC (Fig. 6b), the CPC-induced MitoMP changes as mentioned above recovered to near normal levels compared to CPC only and untreated controls.

### Assessment of γH2AX/PI-based DNA damage of CPC and the effect of NAC pretreatment

**Fig. 7:**
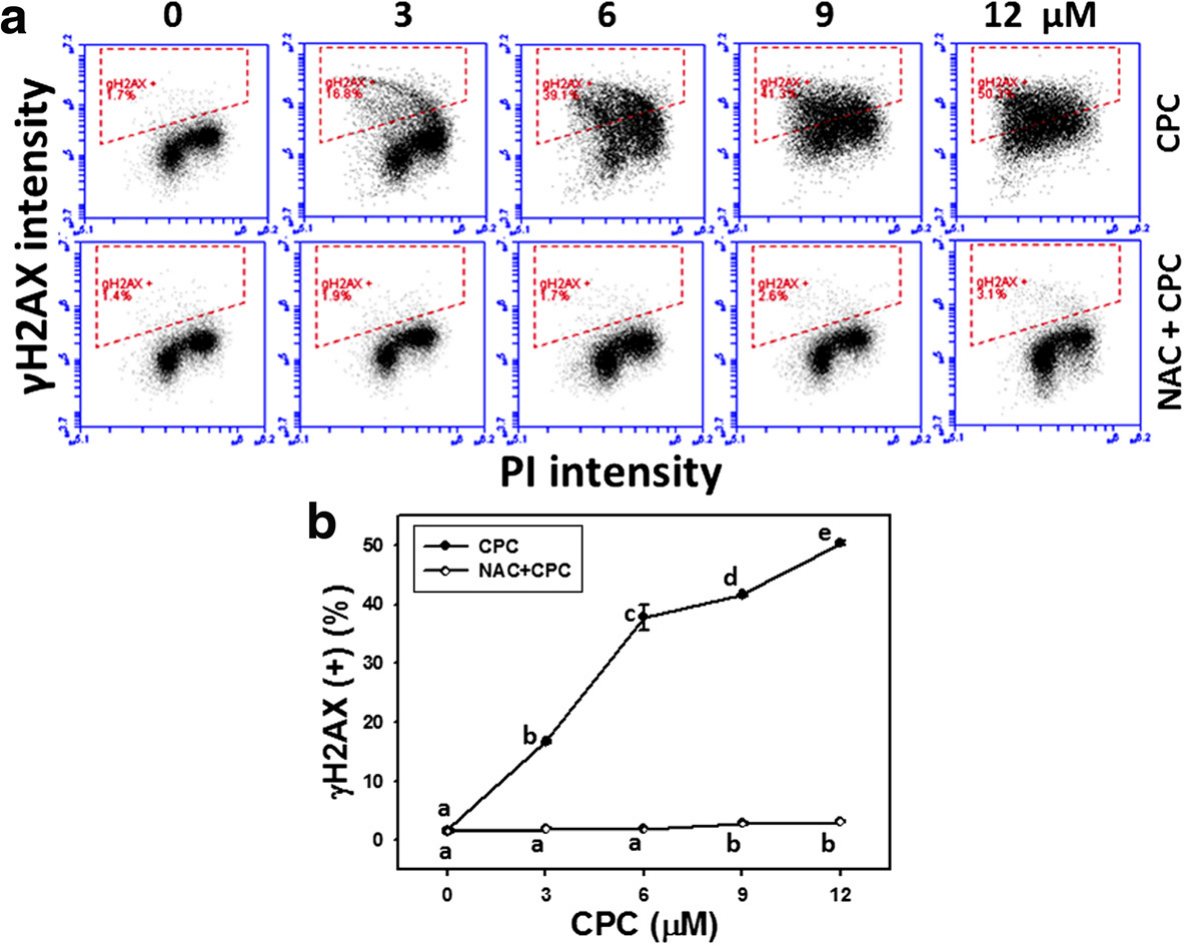
γH2AX-based DNA damage in CPC-treated oral cancer Ca9-22 cells and the effect of NAC pretreatment. With or without 2 mM NAC pretreatment for 1 h, cells were treated with 0, 3, 6, 9, and 12 μM of CPC for 24 h. **a** Representative flow cytometry-based DSB profile for CPC-treated Ca9-22 cells. Dashed lines indicate the γH2AX positive regions in each panel. **b** Quantification analysis of fold changes in γH2AX-based DNA damage in CPC-treated Ca9-22 cells. Data, mean ± SD (*n* = 3). For the same drug treatment of different concentrations, data marks (a to e) without overlapping by the same lower-case letter significantly differed (one-way ANOVA with Tukey HSD Post Hoc Test)

To further examine the role of DNA damage in CPC-induced effects, the DNA double strand break (DSB) marker (γH2AX) was chosen for use in flow cytometry. Figure 7a displays the γH2AX/PI staining profiles of CPC-treated Ca9-22 cells at 24 h incubation. After CPC treatment, the γH2AX-positive staining of CPC (0–12 μM)-treated Ca9-22 cells were dose-responsively decreased (*P* < 0.001) (Fig. 7b).

The γH2AX staining profiles of NAC pretreatment effect against CPC-induced DNA damage were also demonstrated in Fig. 7a (bottom). After NAC pretreatment, i.e., NAC + CPC (Fig. 7b), the CPC-induced γH2AX changes as mentioned above recovered to near normal levels compared to CPC only and untreated controls.

## Discussion

*Cryptocarya*-derived compounds were identified has having diverse biological functions including antiproliferation for several types of cancer. However, its effect on oral cancer cells has been less-well addressed. The current study examines the possible anti-oral cancer effect of *C. concinna*-derived CPC and explores drug response mechanism in detail.

### Selective killing of CPC

Several anti-oral cancer drugs have harmful side effects for normal cells, thus limiting their clinical applications, and anticancer therapies ideally should have selective cell killing effects [21, 37]. In current study, we found that CPC were cytotoxic to two oral cancer cells but less harmful to oral normal cells (Fig. 1). Accordingly, CPC has the potential for selective killing to oral cancer cells.

### Comparison of drug sensitivity of CPC

Cryptocaryone has recently been reported to inhibit proliferation of prostate cancer cells where its IC_50_ value is 1.6 to 3.4 μM 48 h by SRB assay [19]. *C. konishii*-dervied cryptocaryone was found to inhibit proliferation of murine leukemia P-388 cells where its IC_50_ value is 0.04 μM 48 h by MTT assay [17]. Cryptocaryone was also reported to be an active compound for antiproliferation in the KB cell lines with IC_50_ values 1.8 μM at 3 days by MTT assay [39]. However, KB cells were recently found to be human cervical cancer HeLa cells rather than oral epidermal carcinoma [40]. Accordingly, the anti-oral cancer effects of cryptocaryone are still largely unknown. In the present study, we firstly found that CPC had an antiproliferative effect against oral cancer cells and the IC_50_ values of CPC in oral cancer Ca9-22 and CAL 27 cells at 24 h by MTS assay were 11.63 and 3.91 μg/ml, respectively.

### The role of apoptosis in CPC studies

The current study provides evidence for the apoptosis effect of CPC in oral cancer Ca9-22 cells, such as subG1 accumulation, annexin V/PI staining, and pancaspase analyses (Figs. 2, 3 and 4). Gene expression of apoptotic and anti-apoptotic proteins may be further validated by western blot or by PCR. For example, we found that apoptotic protein poly (ADP-ribose) polymerase (PARPγ) [41] of Ca9-22 cells and anti-apoptotic protein BCL2 [42] of CAL 27 cells was respectively up- and down-regulated after CPC treatments (data not shown). In future, work related to p53, p21, p27 and phospho p53 will be of more additional value to further find the detailed effect of CPC on apoptotic pathway. Similarly, cryptocaryone also reportedly induced apoptosis in human prostate cancer PC3 cells in terms of the subG1 accumulation, cleavage of caspase-8 and 3, death receptor DR5 accumulation on membranes, and up-regulation of Mcl-1 expression [19]. However, the role of oxidative stress of CPC-treated PC3 cells is not addressed. Instead, we had discussed the involvement of oxidative stress in CPC-treated oral cancer cells in next section.

### The role of oxidative stress in dihydrochalcone studies

CPC is a kind of dihydrochalcone that is shown to kill oral cancer cells (Fig. 1). Similarly, *Muntingia calabura*-derived dihydrochalcones (2′,4′-dihydroxy-3′-methoxydihydrochalcone and (-)-3′-methoxy-2′,4′,beta-trihydroxydihydrochalcone) have been reported to be cytotoxic to murine leukemia P-388 cells and human colon cancer HT-29 cells [43]. *Corema album*-derived dihydrochalcones (2′,4′-dihydroxydihydrochalcone and 2′-methoxy-4′-hydroxydihydrochalcone) have been reported to be cytotoxic to colon cancer HT-29 cells [20] and their cell killing effects were reduced by NAC pretreatment [20], Consistently, we found that NAC pretreatment can inhibit CPC-induced ROS generation (Fig. 5) and mitochondrial depolarization (Fig. 6). Moreover, we also found that the CPC-induced subG1 accumulation, apoptosis (annexin V and caspase activities), and DNA damage were rescued by NAC pretreatment (Figs. 2, 3, 4 and 7). These findings suggest that oxidative stress may be involved in the cytotoxic activity of these dihydrochalcones. Moreover, oxidative stress may induce autophagy and apoptosis [44]. In future, it warrants for modulating autophagy and apoptosis by inhibitors such as 3-methyladenine/chloroquine [45] and Z-VAD-FMK [46] to investigate their individual contributions on CPC-induced antiproliferation of oral cancer cells.

However, some dihydrochalcones may have different cell responses. For example, neohesperidin dihydrochalcone, a non-nutritive sweetening agent produced by hydrogenation of neohesperidin, displayed antioxidant properties to inhibit hypochlorous acid-induced DNA damage and cell death [47] and to inhibit carbon tetrachloride-induced oxidative damage both *in vivo* and *in vitro* [48]. Therefore, the functions of dihydrochalcones may have dual roles and may be dependent on its chemical properties, i.e., ROS generation or scavenging.

## Conclusion

**Fig. 8:**
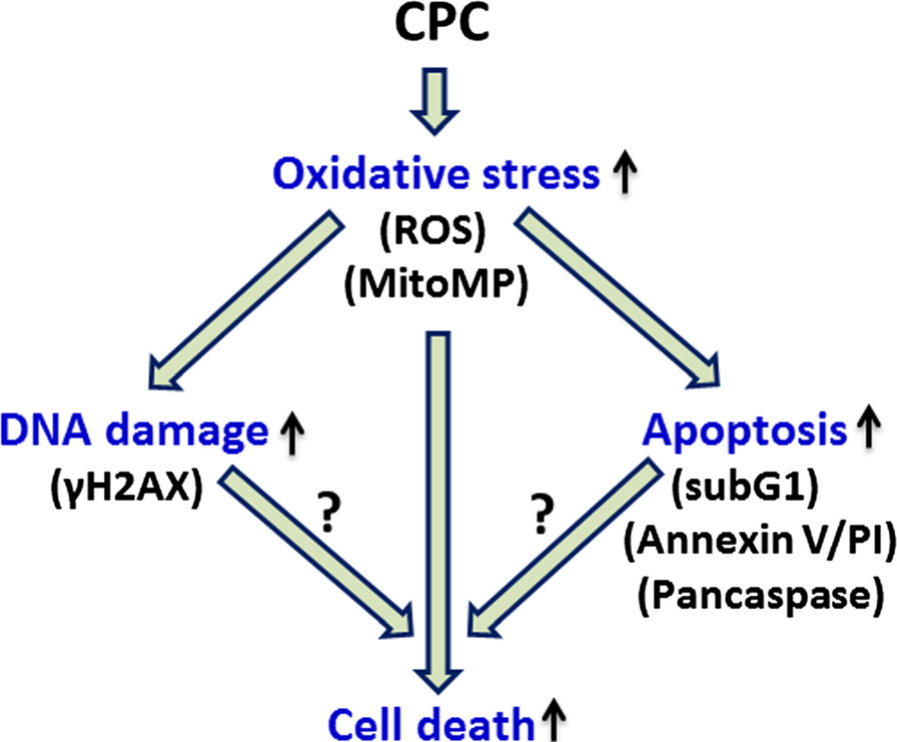
Schematic diagram of hypothesized mechanism of CPC-induced cell killing of human oral cancer cells. Changes in oral cancer cells (Ca9-22) are indicated by bold arrows, such as increases of oxidative stress, apoptosis, DNA damage, and cell death. The upward arrows indicate the increase in each assay of this study. Different assays were shown in parentheses. The bold arrow with “?” symbol is not examined in this study

In conclusion, we demonstrate that CPC can inhibit cell proliferation and apoptosis of oral cancer cells but is less harmful to normal oral cells. This cell killing mechanism includes the ROS generation, mitochondrial depolarization, and DNA damage, which can be rescued by NAC pretreatment. Therefore, these results suggest that CPC has an anticancer potential for oxidative stress-mediated oral cancer therapy based on the cell line study (Fig. 8). In future, *in vivo* methods to explore the *in vivo* mechanism will provide concrete evidence of CPC.

## Additional file

Additional file 1: Figure S1.
^1^H NMR and ^13^C NMR spectrums of CPC. (DOCX 151 kb)
