# Supplementary material for: Antiproliferation of Cryptocarya concinna-derived cryptocaryone against oral cancer cells involving apoptosis, oxidative stress, and DNA damage
Source: BMC Complement Altern Med. 2016 Mar 8;16:94. doi: 10.1186/s12906-016-1073-5 (PMC4784356; doi:10.1186/s12906-016-1073-5)
Supplement: Additional file 1: Figure S1. — 1H NMR and 13C NMR spectrums of CPC. (DOCX 151 kb) [file 12906_2016_1073_MOESM1_ESM.docx]

**Additional file 1:**

**Figure S1. ^1^H NMR and ^13^C NMR spectrums of CPC.**

**(A) ^1^H NMR (CDCl_3_ 400 MHz):** δ (ppm). 2.61 (1H, dd, *J* = 17.6, 12.4 Hz, H-11a), 2.78 (1H, dd, *J* = 17.6, 8.8 Hz, H-11b), 4.00 (1H, m, H-5), 5.47 (1H, dt, *J* = 8.8, 2.0 Hz, H-6), 6.20 (1H, dd, *J* = 10.4, 2.0 Hz, H-8), 6.54 (1H, dd, *J* = 10.4, 2.0 Hz, H-7), 6.78 (1H, d, *J* = 15.6 Hz, H-3), 7.42 (3H, m, H-3′, H-4′, and H-5′), 7.56 (2H, m, H-2′ and H-6′), 7.76 (1H, d, *J* = 15.6 Hz, H-2).

**(B) ^13^C NMR (CDCl_3_ 100 MHz):** δ (ppm) 34.0 (C-5), 35.3 (C-11), 76.1 (C-6), 103.3 (C-10), 116.6 (C-3), 128.2 (C-2′ and C-6′), 129.1 (C-3′and C-5′), 130.1 (C-4′), 130.6 (C-8), 134.8 (C-1′), 139.8 (C-7), 142.4 (C-2), 174.1 (C-4), 174.4 (C-12), 185.8 (C-9)
